# P70 Improving safety and efficiency of biologic and DMARD therapy prescribing through development of a multi-disciplinary virtual clinic

**DOI:** 10.1093/rap/rkac067.070

**Published:** 2022-09-28

**Authors:** Jasper Street, Gavin Pinington, Rachael Derbyshire, Emily Willis

**Affiliations:** Royal Manchester Children's Hospital, Manchester, United Kingdom; Manchester University NHS Foundation Trust, Manchester, United Kingdom; Royal Manchester Children's Hospital, Manchester, United Kingdom; Royal Manchester Children's Hospital, Manchester, United Kingdom

## Abstract

**Introduction/Background:**

We identified that the system for prescribing biologic and DMARD therapies in paediatric rheumatology at Royal Manchester Children’s Hospital (RMCH) was slow, often causing repetition of work and delays in timely access to repeat and new prescriptions. We created a multidisciplinary team (MDT) virtual clinic involving specialist nurse, pharmacist and consultant to allow for an efficient and safe “one-stop shop” process to review all repeat and new prescriptions. The aim of the VBC was to improve efficiency and communication around prescribing, reduce time to commence new therapies, and ensure more robust oversight of therapeutic drug monitoring and patient safety.

**Description/Method:**

Approximately 400 patients currently receive prescriptions via homecare systems for subcutaneous biologics and disease modifying antirheumatic drugs (DMARDS) under the care of the RMCH paediatric rheumatology service. These patients require close monitoring and timely repeat prescribing to ensure safe management.

Historically, a prescription request list was sent from pharmacy homecare team to rheumatology specialist nurses, who would review patient records, print prescriptions, and organise a weekly meeting with the responsible consultant to review and sign the prescription. Prescriptions hand delivered to the pharmacy homecare office would await pharmacist verification and submission to the homecare provider. Any pharmacist queries emailed to the nursing team required review, and queries would need to be dealt with by nursing or medical staff, introducing multiple opportunities for delay into the prescribing process. This multi-step process created numerous hours of administrative burden due to silo-working and poor communication.

Rheumatology nursing, pharmacy and medical personnel worked together to secure funding approval for a new virtual biologics clinic (VBC), where repeat and new prescriptions would be reviewed weekly by these core team members. For each patient, the diagnosis, indication for therapy, relevant blood results, clinical parameters (e.g. age, weight) and other pertinent factors are reviewed. If screening identifies no barriers to supply, the prescription is signed by the consultant, checked and countersigned by the pharmacist, who submits completed prescriptions to the pharmacy homecare team the same day. BlueTeq forms are completed by the pharmacist or consultant during the VBC, reducing funding risk and ensuring national commissioning framework requirements are followed.

Where issues are identified e.g. inadequate screening prior to starting biologics or abnormal blood results, management plans can be formulated and actioned in a timely manner.

A clinic letter is generated for each patient discussed, informing the patient, GP and consultant of the outcome of the meeting and actions.

**Discussion/Results:**

The first VBC took place in February 2020 and remains a weekly occurrence. The clinic takes approximately one hour to complete with around two hours of preparation time (checking relevant results and generating prescriptions). An average of 15 – 30 patients are discussed each week. To our knowledge we are the only UK paediatric rheumatology centre to run a VBC in this manner.

Initial staff perception has been that therapeutic optimisation has been achieved in an efficient, timely manner. Consideration of updated weight and age at each review has allowed doses to be increased and identified opportunities for patients to switch to more suitable or better tolerated devices (e.g. moving from etanercept vials to pre-filled syringes or pre-filled pens).

MDT working, sharing of skills and knowledge, has also made it easier to update prescribing guidelines and standardise practice.

Prescription generation prior to the VBC has allowed a move from a reactive to proactive approach. Forecasting of when new prescriptions are due ensures that prescriptions are signed (and any issues dealt with) before request from the homecare provider, reducing the risk of patients running out of medicines and unintended breaks to treatment.

Improved communication within the MDT prevents duplication of work, enabling time savings to carry out other duties.

The clinic letter generated ensures that families know their child’s prescription has been issued, along with communication around changes to therapy, which has reduced telephone calls to the department.

The VBC rollout coincided with the RMCH biosimilar switch of adalimumab. Expedition of transition to a biosimilar improved Trust cost savings. The VBC facilitated the introduction of use of subcutaneous tocilizumab, reducing need for day-case attendance and reducing use of intravenous tocilizumab which was in demand as a COVID-19 rescue medicine.

**Key learning points/Conclusion:**

The VBC has improved efficiency of prescribing, facilitated robust monitoring of patients and has streamlined the process of prescribing, freeing up time for staff to complete other duties.

An additional, unanticipated benefit has been the recognition of safeguarding concerns. Through reviewing each patient’s record before issuing prescriptions, we have identified patients who have been lost to follow-up (a particular concern since the COVID-19 pandemic) or repeated non-attendance at appointments. Early recognition has allowed rapid escalation and resolution of issues.

Completing BlueTeq requests during the VBC has reduced delays in initiation of therapies and minimised NHS England funding challenges. Consultants have an improved understanding around commissioning criteria and alternative funding options, should NHS funding not be available for certain indications and patient cohorts.

We continue to evaluate the VBC. We plan to audit the service, collecting data on time from decision to commence a treatment to starting the new medication, as this is an area we feel has significantly improved with the introduction of the VBC. It is essential to gain feedback from patients and families on their experience of commencing treatments, ease of obtaining repeat prescriptions from homecare and communication of therapy changes.

All three members of the MDT are vital for the efficient running of the VBC and all contribute different expertise. At present the same consultant attends each VBC, future developments may be to rotate this among all the consultants in our team. We could invite trainees to attend, as the clinic provides significant learning opportunities including reviewing indications for immunosuppressant therapy, safe prescribing and interpretation of blood results.

As the Trust moves to an electronic prescribing system in autumn 2022, it is hoped that the prescribing system will become even more streamlined, allowing for electronic signatures and consolidating systems to ensure data is easily accessible.

